# The Association Between Independent Study Desk Ownership and Borderline Personality Features in Children and Adolescents in Difficulty: Findings from a Cross-Sectional Census in China

**DOI:** 10.3390/bs16060836

**Published:** 2026-05-22

**Authors:** Ling Chen, Mingzi Ma, Jiawen Wu, Changyu Fan

**Affiliations:** 1 School of Law and Economics, Wuhan University of Science and Technology, Wuhan 430065, China; chenlingccnu@126.com; 2 School of Sociology, Central China Normal University, Wuhan 430079, China

**Keywords:** children in difficulty, borderline personality features, family educational resources, social support

## Abstract

Children in difficulty are at heightened risk of developing personality-related problems, partly due to gaps in family care and social protection. This study examines whether (a) the availability of an individual study desk at home—a basic family educational resource—is associated with borderline personality features (BPFs) in this population and (b) perceived social support and school life shape this association. Using survey data from 14,993 children in difficulty in Hubei Province, China, and applying propensity score matching (PSM), we find that children with their own desk report significantly lower levels of BPFs. The protective association between desk ownership and children’s perceptions of social support is stronger when children perceive higher social support, whereas indicators of school life do not significantly moderate this relationship. Heterogeneity analyses further show that the benefit of having a desk is greater for children without siblings. These findings highlight the protective role of physical learning environments and suggest that targeted provision of basic educational resources could serve as a practical entry point for early-intervention programs aimed at reducing the risk of developing BPFs.

## 1. Introduction

Children in difficult circumstances have received increasing global attention as a particularly vulnerable population requiring targeted mental health research and intervention. This group broadly includes orphans, children at high risk of HIV infection, children with severe disabilities or serious illnesses, children living in extreme poverty, and children without adequate care (Sheehan, 2010; Collins & Wright, 2022; Cannon et al., 2019). In the Chinese policy context, children in difficult circumstances have been defined as those who require assistance or protection because of adversity related to individual, family, or external factors. This definition includes children facing difficulties in daily living, medical care, or education due to family poverty; children with disabilities who require support in rehabilitation, care, nursing, or social integration; and children whose safety is threatened or violated because of abuse, neglect, accidental injury, unlawful infringement, or inadequate family guardianship (General Office of the State Council, 2025).

Compared with their peers, these children are at an increased risk of experiencing feelings of worthlessness, social isolation, and frustration, primarily due to limited access to effective family care and social protection (Bamba & Haight, 2011). Specifically, chronic life challenges among children in difficulty may disrupt the development of social–cognitive mechanisms and lead to biased patterns of social information processing, such as hyper-vigilance to rejection or distorted interpretations of social cues (Dodge et al., 1994; Lutfey & Mortimer, 2006). These socialization deficits are directly associated with core features of borderline personality disorder (BPD), including persistent instability in interpersonal relationships and intense fears of abandonment. Consequently, such negative person–environment interactions foster developmental vulnerabilities that eventually consolidate into borderline personality pathology (Van Os & Jones, 1999; de Groot et al., 2024). Among the various personality disorders, BPD has received considerable research attention due to its severe clinical manifestations. Individuals with BPD often show persistent instability in interpersonal relationships, fears of abandonment, identity disturbance, impulsive behaviors, and marked affective dysregulation, which may include intense anger or anxiety (Lieb et al., 2004; American Psychiatric Association [APA], 2013; Keepers et al., 2024).

While BPD is typically diagnosed in late adolescence or adulthood, some studies suggest that its core features—such as emotional disorders and unstable interpersonal relationships—are already apparent at an earlier stage (Chanen et al., 2017, 2020; Bozzatello et al., 2021). The Diagnostic and Statistical Manual of Mental Disorders (DSM-5) notes that certain types of personality disorder features may be recognized before the age of 18 when maladaptive traits have been present for at least one year and are not limited to a particular developmental stage (American Psychiatric Association [APA], 2013). Given that a formal diagnosis of BPD in childhood remains clinically inappropriate and controversial, researchers have increasingly shifted toward borderline personality features (BPFs) as a sensitive construct to capture the manifestations of borderline pathology, as BPFs strongly predict adult BPD and are substantially shaped by the early family environment (Sharp et al., 2020; Skabeikyte & Barkauskiene, 2021). This shift is particularly important because early identification of BPFs provides valuable opportunities for preventive intervention before the full clinical syndrome consolidates (Chanen et al., 2020).

Borderline personality disorder has been linked to the interaction between genetic factors and adverse environmental experiences (Boucher et al., 2017; Bozzatello et al., 2021). Family-related factors are often considered particularly prominent among the environmental risk factors. Traumatic experiences within the family, such as abuse, neglect, maladaptive parenting styles, and insecure attachment, may predict symptoms of BPD (Harman, 2004; Stepp & Lazarus, 2017; Boucher et al., 2017; Porter et al., 2020). Notably, a meta-analysis by Porter et al. (2020) reported that emotional abuse and neglect show particularly strong associations with BPD symptoms. In addition, the biosocial theory suggests that an invalidating caregiving environment may intensify emotional vulnerability and contribute to maladaptive behaviors (Crowell et al., 2009). Beyond relational and caregiving factors, the broader material conditions of the home environment may also play a role. A lack of material resources, such as overcrowded housing and inadequate living conditions, has been identified as an indicator of adverse developmental environments (Solari & Mare, 2012; Lorentzen et al., 2024). More specifically, research has shown that the home learning environment, encompassing both material resources and parental engagement in learning-related activities, predicts children’s development across cognitive and socioemotional domains, independently of socioeconomic status (Niklas et al., 2016; Niklas & Schneider, 2017; Wirth et al., 2022). The availability of a dedicated learning space may therefore serve as one observable indicator of these broader home environmental conditions. Importantly, the adversities experienced by children in difficulty are rarely unidimensional. According to the theory of intersectionality introduced by Kimberlé Crenshaw, the convergence of multiple marginalized identities, such as extreme poverty, lack of adequate guardianship, or physical disability, does not merely result in an additive burden of oppression (Crenshaw, 1989). Instead, it creates a unique and qualitative ‘complex of oppression’ where different vectors of disadvantage interact to shape a distinct experience of vulnerability. In this sense, a child facing multiple systemic barriers experiences not simply a compounded version of individual hardships but rather a unique, multi-vectored form of psychological and social strain. By examining the availability of an independent study desk, this study seeks to understand whether a micro-level educational resource may function as one observable marker associated with the complex, intertwined risks that shape the personality development of these children. While genetic and clinical factors are central to BPD research, the present study adopts a social science perspective and focuses on BPFs as a dimensional, nonclinical construct measurable in community child samples.

Beyond family factors, the impact of social environment has been of increasing concern in recent years. Studies suggest that peer bullying and social exclusion during childhood are important predictors of BPD symptoms and that being bullied may increase the risk of developing BPD symptoms (Wolke et al., 2012; Winsper et al., 2017). These findings emphasize the importance of examining the family factors and broader social background when studying the development of BPFs.

Although progress has been made in understanding the risk factors for BPD in Western populations, research on BPD/BPFs among Chinese children remains limited. Cross-cultural research is essential because cultural context may shape the expression of borderline characteristics and the effects of environmental risk factors (Wu et al., 2022; Yuan et al., 2025). Using provincial survey data from Chinese children in difficult circumstances, this study examines a specific family factor—investment in educational material resources (i.e., the availability of an independent study desk)—and its association with BPFs. It also investigates whether broader social factors, including perceived social support and school life, influence this relationship.

## 2. Research Hypothesis

### 2.1. Borderline Personality Features in Youth

#### 2.1.1. Borderline Personality Features in Children and Adolescents

Borderline personality features refer to enduring patterns of behavior and inner experience that typically emerge in adolescence or early adulthood, remain relatively stable over time, and are associated with significant functional impairment and psychological distress (American Psychiatric Association [APA], 2013). Borderline personality disorder represents one of the most frequently diagnosed personality disorders in clinical settings (O’Dwyer et al., 2020). Due to the stigma surrounding the diagnosis, clinicians have historically been reluctant to assign a formal BPD diagnosis to individuals under the age of 18, even when a substantial proportion of younger patients meet the diagnostic criteria (Østby et al., 2014). Consequently, BPFs and their associated risk factors have attracted growing attention among clinicians and service providers as a more pragmatic framework for identifying and addressing maladaptive traits in children and adolescents. Further complicating the picture, BPD arises from a complex interplay of biological and psychosocial risk factors, with no single causal pathway and considerable heterogeneity across individuals (Paris, 2014). Targeting subclinical BPFs therefore represents a more clinically actionable approach than awaiting a formal BPD diagnosis.

Drawing on Linehan’s biosocial theory, Crowell et al. (2009) proposed a biosocial developmental model of borderline personality, positing that BPD originates in early constitutional vulnerabilities (most notably impulsivity) followed by heightened emotional sensitivity. Over the course of development, these vulnerabilities are amplified by environmental risk factors, ultimately giving rise to increasingly severe dysregulation across emotional, behavioral, and cognitive domains. Clinical and empirical evidence consistently links adult BPD to a range of developmental antecedents. The disorder is frequently preceded by adverse childhood experiences, including disrupted attachment relationships, maladaptive parenting, maternal neglect and rejection, abuse, and familial psychopathology (Ludolph et al., 1990). Adolescent Axis I conditions have also been identified as notable precursors among individuals later diagnosed with personality disorders. That said, a BPD diagnosis in adolescence does not necessarily persist into young adulthood (Bernstein et al., 1993; Sharp et al., 2014). Although the developmental trajectory of BPFs in early adolescence is heterogeneous, the broader evidence indicates that elevated BPD symptomatology is prospectively associated with a range of adverse outcomes, including poorer psychosocial functioning, increased sexual risk-taking, reduced attainment of adult role functioning, diminished social functioning and life satisfaction, lower educational and occupational achievement, and less stable romantic partnerships (Carreiras et al., 2022).

#### 2.1.2. The Family Learning Environment as a Critical Antecedent of Borderline Personality Features

According to the biosocial developmental model, the emergence of BPFs results from the continuous interaction between an individual’s constitutional vulnerabilities and their surrounding environmental conditions. Borderline personality features are believed to be closely associated with multiple family environmental risks, particularly long-term, cumulative environmental instability and impaired caregiving processes. Usually, the physical environment of a family is seen as a direct reflection of socioeconomic status (SES). Children from low SES backgrounds often face physical environments that are overcrowded, noisy, chaotic, and lack educational resources (Evans et al., 2010; Solari & Mare, 2012). Within this biosocial framework, resource conservation theory (Hobfoll, 1989) provides a mechanism for understanding how such environmental adversity functions by emphasizing the depletion of individual resources as a primary stressor. This kind of chaotic household environment, characterized by high noise level, lack of order, and unpredictability, can weaken children’s self-regulation and ability to cope with stress and is significantly associated with social emotional difficulties, behavioral problems, and psychological distress (Matheny et al., 1995; Marsh et al., 2020; Mitchell et al., 2025). In this sense, the living environment may shape children’s sense of security and perceived control over self-identity through chronic stress exposure and constrained day-to-day interactions (Midouhas et al., 2026; Fouts et al., 2017).

However, the influence of the physical conditions of the household is not equivalent to SES determinism. Research has shown that crowding and chaos often have an indirect impact on children’s development by weakening parents’ responsiveness and the quality of daily care (Evans et al., 2010). This complexity is further highlighted by the intersectional perspective, which suggests that enduring stress in these children arises from a multi-vectored environment where overlapping disadvantages reinforce one another, resulting in a systemic vulnerability that is more than the sum of its parts (Crenshaw, 1989). Parents may construct an environment that promotes development if they allocate learning-related resources, organize daily activities, and provide interaction opportunities for their children (Guo & Harris, 2000; Bradley & Corwyn, 2002). Even in resource-constrained families, active parental involvement and environmental reorganization can offset some adverse effects and serve a buffering role (Evans & Kim, 2013). Material resources and activity spaces for children’s learning, as well as parent–child activities centered around learning, are highly correlated with children’s language, academic, and some social-emotional outcomes, even after controlling for SES (Melhuish et al., 2008).

Consistent with the biosocial model’s emphasis on protective environmental factors, a stable and organized learning space serves as a tangible proxy for the family’s broader developmental support, which is hypothesized to buffer the negative effects of environmental stress on the development of BPFs. Privacy, opportunities for retreat, and a sense of control over the environment are considered important foundations for mental health and autonomous development (Evans, 2006; Vlasova et al., 2020). For children, having a space of their own may support self-regulation, agency, and the development of self-identity (Korpela et al., 2002). The biosocial theory further elucidates how these physical boundaries facilitate psychological growth, as a coherent sense of self partly derives from early experiences in which boundaries, both psychological and physical, are respected and maintained (Mahler et al., 1975). This alignment is crucial since a core trait of BPFs is identity instability and difficulties with interpersonal boundaries (American Psychiatric Association [APA], 2013).

However, the presence of independent learning space is not a standalone indicator of a supportive environment. In some contexts, it may reflect excessive academic expectations that impose psychological stress on vulnerable children (Lu et al., 2021; Xu et al., 2024). Within the biosocial framework, the efficacy of this resource is contingent upon the quality of familial interactions and whether the child’s autonomy within that space is respected. For a learning environment to be truly supportive, it must move beyond physical infrastructure to include a responsive and flexible climate that acknowledges the diverse needs and cultural contexts of the child, ensuring that the desk functions as a site of empowerment rather than merely a tool for academic compliance. In summary, the family’s physical environment, family interaction processes, and children’s boundary experiences may represent multiple pathways related to BPFs. To address the limitation that existing research often relies on measures that are difficult to obtain quickly in practical contexts, this study examines whether an easily observable family cue—that is, whether a child has a fixed, independent study desk—can serve as a proxy indicator of these family conditions. While an independent desk or fixed learning space does not determine personality development, it may function as a low-cost, observable marker of family resources, household organization, learning support, and respect for children’s autonomy and boundaries. Ultimately, the use of this proxy is consistent with the biosocial model of BPD, which emphasizes interactions between biological vulnerability and environmental risk and protective factors (Crowell et al., 2009). Consequently, this paper posits the following:

**Hypothesis** **1.** *The presence of a study desk is negatively associated with BPF among children in difficulty.*

### 2.2. Social Support and Its Protective Association with Borderline Personality Features

Social support encompasses the positive material, emotional, and informational assistance that individuals receive from their social networks, including family, relatives, friends, leaders, and colleagues, particularly during challenging circumstances (Sarason et al., 1983; Sarros & Sarros, 1992; Steele & Lynch, 2013). This support can be categorized into two primary forms: emotional and instrumental. Both types positively contribute to an individual’s mental health by facilitating information exchange and fostering specific interpersonal relationships (Finfgeld-Connett, 2005). The buffer model theory of social support posits that social support serves as a moderator for various factors that may adversely impact an individual’s physical and mental well-being (S. Cohen & Wills, 1985). Specifically, in times of stress, social support can diminish the perceived intensity of stress, thereby alleviating its detrimental effects on both physical and mental health (Miloseva et al., 2017). Empirical evidence indicates that familial social support enhances children’s self-esteem and self-confidence and provides a stable emotional foundation that enables them to effectively navigate life stressors, thereby mitigating potential negative effects of stressors on their mental health, such as BPFs (Holahan et al., 1995).

Furthermore, social cohesion within neighborhoods significantly influences children’s mental health (Sampson, 2003), whereas inadequate neighborhood relationships can exacerbate the behavioral and cognitive issues of children in difficulty (Choi et al., 2018). The effects of social support extend beyond children’s mental health and BPFs; social support also critically affects families’ access to educational resources. Research indicates that children may face educational disadvantages in communities that lack sufficient support (Evans & Kantrowitz, 2002). Additionally, perceived social support is vital for fostering positive parenting practices and facilitating child development, thereby creating a nurturing home environment conducive to children’s growth (Jackson et al., 2000). However, children in difficulty often experience a scarcity of social support, which is predominantly sourced from within their families (Nevard et al., 2021). These children frequently encounter challenges in obtaining adequate social support (Killian & Durrheim, 2008), resulting in a diminished quality of life and increased psychological vulnerability. Notably, individuals with BPFs often find themselves at a disadvantage in social contexts, with the severity of their symptoms closely linked to their perceived lack of social support (Bijttebier & Vertommen, 1999; Beeney et al., 2018). Consequently, the effective provision of social support is crucial for assisting these children in accessing essential educational resources, alleviating stress, and enhancing their mental health. This leads to the formulation of Hypothesis 2:

**Hypothesis** **2.** *Social support moderates the association between having a separate desk and BPFs in children with difficulties.*

Schools serve not only as vital settings for adolescent cognitive development but also as essential environments for fostering positive social relationships and emotional behaviors (Wilson, 2004; J. Cohen et al., 2009). School climate, which refers to the overall culture and environment of a school, is characterized by its enduring and stable nature, significantly influencing the psychological well-being and behavior of its members (Hoy & Hannum, 1997). According to the stage–environment matching theory (Eccles, 2004), the core psychological needs of children, including relationships, autonomy, and competence, are fundamental to their development. A school climate that effectively addresses these needs can be crucial in facilitating positive growth during adolescence. Additionally, the interpersonal dynamics within the school environment exert a direct and substantial influence on students’ mental health. Self-determination theory (Deci & Ryan, 1985) posits that adolescents’ fundamental psychological needs—such as security, intimacy, and autonomy—are more likely to be fulfilled in a supportive environment (Reeve, 2002; Wang & Dishion, 2012). The quality of relationships within the school context is a significant predictor of students’ psychological adjustment (Wang & Degol, 2016). Positive interactions with teachers and peers and a well-ordered school environment can enhance the self-esteem of children from challenging backgrounds and alleviate adverse psychological symptoms (Way & Robinson, 2003). Furthermore, students’ prosocial behaviors are bolstered when they perceive support from individuals with various roles within the school (Wentzel, 1991). Research indicates that schools that prioritize learning and student autonomy tend to have students who exhibit fewer symptoms of personality disorders (Kasen et al., 2009). A positive school climate encourages students to address negative emotions, mitigates the impact of adverse experiences, and fosters positive self-perceptions (Chan & Wong, 2015). The perceived learning climate is a critical factor influencing not only adolescent BPFs but also overall adolescent physical and mental health (Jia et al., 2009; Wang & Dishion, 2012). Empirical evidence suggests that adolescents’ perceptions of school climate can compensate for deficiencies in educational resources at home (Way et al., 2007). When the school environment is characterized by effective classroom management, consistent teacher support, and harmonious peer relationships, it can stimulate student interest and engagement, thereby promoting active self-development (Eccles & Roeser, 2009). Students who feel safe, valued, and supported within the school context tend to exhibit increased motivation and academic success (J. Cohen et al., 2009). The school climate, particularly the teacher–student relationship, is significant in enhancing children’s social skills and academic performance (Esposito, 1999). Given these considerations, this paper posits the following:

**Hypothesis** **3.** *School life moderates the association between having a study desk and BPF in children with difficulties.*

## 3. Methods

### 3.1. Sampling

Our research is part of an extensive census investigating the psychological well-being of underprivileged children in Hubei Province, China. Data collection was conducted between 7 December and 18 December 2023. This census was supported by the Hubei Provincial Department of Civil Affairs. Before the survey began, the researchers provided brief online training to the child directors (dedicated grassroots officials responsible for children’s and women’s affairs in each village and community) to ensure that they could understand the meaning of the questionnaire and control the quality of the responses as questionnaire distributors and assistants. The questionnaire is mainly filled out by children and caregivers together, and the child directors provide explanations and clarifications to help respondents understand the questions during the process. With the support of the Ministry of Civil Affairs, the researchers ultimately determined the survey samples in each rural or urban community with the assistance of local child directors. The number of respondents in each community ranges from 4 to 10, depending on the number of children in difficulties officially recorded by the government. The census involved 41,982 questionnaires. Following data collection, quality control procedures were implemented to exclude incomplete or randomly completed responses, as identified by response time thresholds and predefined validation items. A total of 34,748 valid responses were retained, yielding an effective response rate of 82.8%. Final samples targes various groups, including left-behind children, migrant children, children with severe illnesses or disabilities, orphans, neglected children, children facing guardianship challenges, children who have experienced abuse from guardians, underage children of incarcerated individuals, children whose parents have abandoned them, and school dropouts. The study received ethical approval from the Research Ethics Committee of Central China Normal University and support from the Children’s Welfare Division of the Hubei Provincial Civil Affairs Department.

Based on the target population group of the research, we selected 20,202 valid target observations from the overall database. Further, we excluded samples without a complete BPF module and preschool-aged children to ensure the quality of responses in the cognitive module, resulting in a final sample of 14,993 valid observations for our analysis.

### 3.2. Measurements

Borderline Personality Features (BPFs): We set BPFS-C as our dependent variable and adopted the Borderline Personality Features Scale for Children (BPFS-C) to measure it (Crick et al., 2005; Haltigan & Vaillancourt, 2016). The BPFS-C is a 24-item scale comprising four dimensions: affective instability, self-harm, negative relationships, and identity problems. The scale was developed to specifically evaluate borderline personality traits among children aged 9 and older (Crick et al., 2005). The BPFS-C uses a 5-point Likert scale, with higher scores indicating greater severity of borderline personality traits. Its Chinses version has shown good cross-cultural equivalence in Chinese samples (Li & Wu, 2018). The overall Cronbach’s coefficient of the scale in the current study was 0.94.

**Desk:** We selected the availability of an independent study desk as the independent variable. We asked the participants the following question to determine whether they had a desk at home: “Do you have a desk at your home?” On the basis of the responses, coded as “yes” (1) or “no” (0), we divided participants into two mutually exclusive groups in preparation for the matching procedures.

**Moderators:** To explore the moderating effects of potential sources of positive support for disadvantaged children, we chose social support and school life as our moderators at the societal and school levels in addition to the family level. Social support was measured by the Social Support Rating Scale (Xiao, 1994), which comprises 7 items and 3 dimensions (subjective support, objective support, and support utilization), with higher scores reflecting a greater level of social support. The overall Cronbach’s coefficient of the scale in the current study was 0.94. School life was assessed by the School Belonging Scale developed by Goodenow and Grady (1993), which was revised for the Chinese version by Liu et al. (2021). The overall Cronbach’s coefficient of the scale in the current study was 0.96.

**Covariates:** In our literature review, we identified certain potential explanatory factors for BPFs at the demographic level. These factors include age (age in years as of 2023), gender, hukou (household registration in rural or urban areas), siblings (the total number of one’s siblings), and individual emotional status (evaluated by the Depression Anxiety Stress Scale; Lovibond & Lovibond, 1995; Cronbach’s alpha = 0.97). The participant family factors included parental relationship, father’s and mother’s levels of education (1 = Primary school or below, 2 = Junior high school, 3 = High school, 4 = Junior college, 5 = Bachelor’s degree, 6 = Master’s degree or above), and caregiver parenting (measured by the Scale of Guardian’s Rearing Patterns of Left-behind Children developed by Gong (2011); Cronbach’s alpha = 0.79).

### 3.3. Analysis Strategy

In this study, we mainly used the propensity score matching (PSM) method to estimate the potential impact of having a desk on BPFs. Identifying robust associations and potential predictors of psychological outcomes is a primary objective of much research in the social sciences. While longitudinal data are the gold standard for establishing causality, the use of a counterfactual framework in cross-sectional research can enhance the validity of identifying such associations by reducing potential selection bias (Morgan & Winship, 2014). Although PSM, a popular matching technique, may not address more general endogeneity issues, its ability to reduce reliance on functional form assumptions has made it a prominent tool for estimating average treatment effects in social science research (Shipman et al., 2017).

In the first step, we performed a propensity score analysis to control for potential selection bias (Rubin, 1974). The PSM method uses the language of experiments, such as treatment group and control group. In our analysis, the “control” group comprised individuals with no desk at home and the “treatment” group consisted of individuals with a desk at home. Propensity score matching was used to estimate each individual’s probability of treatment assignment based on the observed covariates. Then, we selected only matched sets of treatment and control cases that contained individuals with equivalent values for these predicted probabilities for further analysis. We used a developed PSM package, psmatch2, which is available in Stata 17.0, to estimate the average treatment effect on the treated participants. We adopted a 1:1 matching strategy with replacement, estimated the *p*-score by a logit model, and set the default caliper. The variables used for matching included all of the covariates above, including the moderating variable. Only the sample in common support was matched. All variables were standardized.

We checked the quality of matching before the outcome estimation. In addition, we employed other matching methods (nearest-neighbor matching, kernel matching, and local linear matching) as robustness checks for the results. The matching methods differ in how the treatment assignment was estimated. Nearest-neighbor matching selected the closest observation within the no-desk group on the basis of distance metrics. The Mahalanobis distance was used for distance calculation, and the samples precisely matched the age of the participants in this study. Kernel matching is a nonparametric method that matches by weighting have-used and have-not-used net group characteristics, with weights representing the similarity between the groups. Local linear matching extends the kernel estimator, running a weighted regression for each observation with no digital divide using the digital divide group data. The weights are as above but include a linear term in the weighting function, which helps avoid bias.

In the second step, we examined the possibility that the four potential positive support sources acted as moderators on the relationship between having a desk and BPFs. In the multiple regression analysis, the control variables were consistent between regression and matching and all variables were standardized. Finally, we conducted heterogeneity tests to check whether the causal effect differed in different groups.

## 4. Results

### 4.1. Descriptive Statistics

The total sample comprised 14,993 observations. Before matching, children from countryside backgrounds formed a larger proportion of the “without desk” group (88.09%) compared with the “with desk” group (80.60%). The gender distribution was nearly balanced. However, variations occurred in parental education, with lower education levels reported for those without desks. Participants without desks had more siblings on average (0.78) than those with desks (0.66). Additionally, compared with participants without desks, caregiver parenting and parental relationship scores were generally higher among respondents with desks, suggesting better family support and relationships. Adolescents with desks at home also seemed to perceive more support from social networks, showing a lower level of distress (1.26) compared with those without desks (1.41). After matching, there was no difference except for the outcome variable (see Table 1).

### 4.2. Multivariate Results

First, we checked the quality of PSM according to the recommended procedures. In matched groups, we tested the normalized biases of the variables that were less than 10% (Table 2). Most variables did not reject the null hypothesis test that there was no systematic difference between the treatment and control groups, showing good quality matching. Additionally, no more than 0.79% of observations were off-support; therefore, a few samples were dropped during the matching.

Table 3 presents the results of the PSM and shows the average treatment effect of having a desk on the level of adolescents’ BPFs. The estimated treatment effect indicates that having a desk reduces adolescents’ BPFs by an average of 0.113 points. This result is statistically significant (*p* < 0.001). We used a resampling method to estimate standard error, reinforcing the reliability of these findings. Thus, Hypothesis 1 was supported.

The results from the other three matching methods generally maintained their statistical significance, indicating that our findings were quite robust (Table 4).

### 4.3. Moderating Effect

Before conducting the multiple regression, to control for heteroskedasticity, we tested for homoskedasticity using the Breusch–Pagan/Cook–Weisberg test, which indicated potential heteroskedasticity. The Shapiro–Wilk test showed that some variables were not distributed normally. Therefore, we used the least squares method to test the structural models and adjusted the standard errors of coefficient estimates. Additionally, we checked for potential multicollinearity issues by computing the variance inflation factor. The results for the mean variance inflation factor ranged between 1.37 and 1.84, which were lower values than those that would suggest any relevant multicollinearity issue.

**Figure 1:**
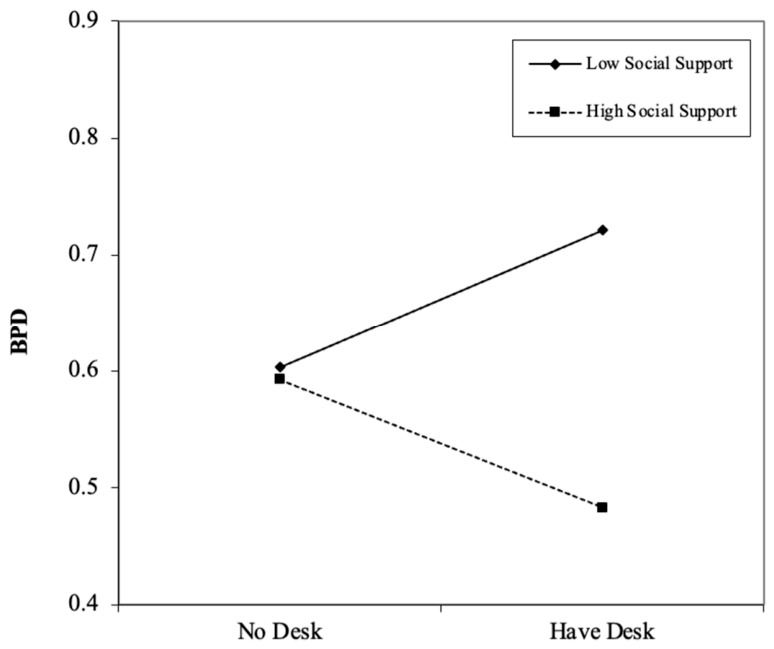
Moderating effects of social support.

Next, we tested the moderating effects of two potential positive support sources for disadvantaged children and adolescents by adding the interactions into our multiple regression models (see Table 5). Our goal was to further investigate the boundary conditions under which having a desk was associated with adolescents’ BPFs. Specifically, Figure 1 shows that social support strengthened the association between desks and borderline personality traits (Model 2, β = −0.043, *p* < 0.001). Thus, Hypothesis 2 was supported. Although we anticipated that school life would influence the effects of desk ownership on BPFs, the results were not statistically significant. Thus, Hypothesis 3 was not supported.

### 4.4. Heterogeneity Analysis

**Figure 2:**
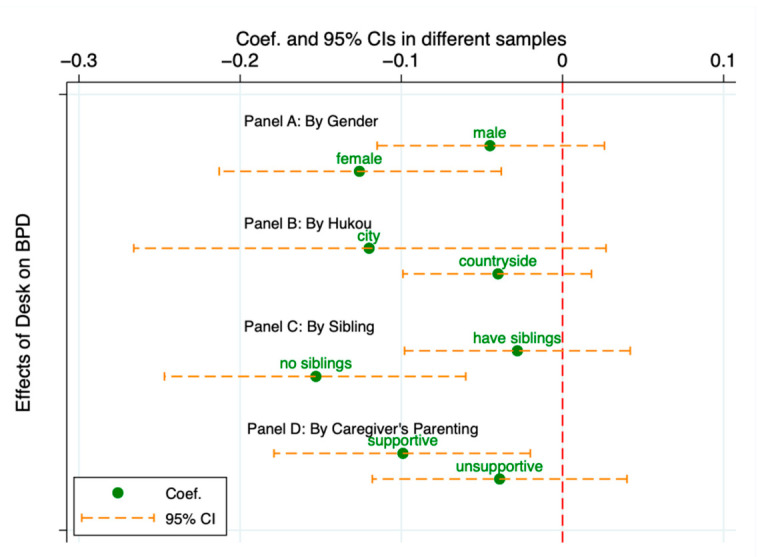
Heterogeneity test results for the estimation effect of desk on BPFs using propensity score matching.

We grouped the samples by gender, hukou (household registration), siblings, and caregiver parenting to examine the heterogeneity of estimated associations. Specifically, we divided the groups into boys and girls, city and countryside samples, samples with and without siblings, and samples with supportive and unsupportive parenting. Next, we calculated the estimated associations for different groups of samples through PSM (Figure 2). We used weighted least squares as a usual method for linear regression to replicate the PSM results and compare the differences between the coefficients. The results show a statistically significant difference between the coefficients of the two groups with and without siblings (*p* = 0.006): compared with respondents with siblings at home, for the sample group without siblings at home, having a desk was associated with a greater reduction in BPF levels.

## 5. Discussion

This study provides associational evidence that the availability of an individual study desk at home is linked to lower levels of BPFs among children in difficulty (Hypothesis 1 supported). The availability of a study desk is more likely to indicate a broader developmental environment rather than a direct driving factor for edge pathology. We interpret a desk as an observable marker/representative of a range of family conditions that may occur in conjunction with children’s psychological development, such as educational material resources, crowding and order, daily life, and learning support.

This finding focuses on the group of children in difficulty whose home educational resources may be significantly associated with their personality development and suggests a potentially meaningful direction for future research on borderline personality in underserved populations. The findings may provide a basis for policymakers to support the promotion of the provision of learning resources, such as desks, in schools and communities to improve children’s mental health. This study contributes to a growing body of work examining associations between the home physical environment and children’s mental health outcomes. A dedicated learning space may indicate that a family is able (and willing) to allocate tangible, learning-related resources and maintain a certain level of structure in daily life, which may be particularly important for disadvantaged children who frequently experience overlapping and cumulative adversity (Melhuish et al., 2008; Evans & Kim, 2013). However, for some families, this may also coincide with higher academic expectations and pressure; moreover, having a desk does not necessarily imply that children have greater agency or that they are the focus of family support. Therefore, the current findings are best interpreted as a preliminary indication and should be extended through more detailed research examining when and how home physical spaces and family interaction processes function as protective environments to reduce the possibility of BPFs.

In addition, we elucidated the context in which an independent study desk may be more strongly associated with BPFs—social support moderates the relationship (Hypothesis 2 supported), school life does not (Hypothesis 3 not supported). This study highlights the need to improve the situation of children in difficulty through social support. For children in difficulty, family distress has been linked to adverse outcomes across multiple domains, including cognitive and emotional development. However, this impact can be moderated through family care and external support systems (Bradley & Corwyn, 2002). Consistent with the buffering effect model of social support (S. Cohen & Wills, 1985), the support of social relationships can attenuate children’s subjective appraisal of the lack of family educational resources, provide them with opportunities for emotional catharsis, partly buffer the detrimental effects of the lack of family educational resources on their socialization, and significantly enhance their integration into the community.

Next, this study meticulously explored the mechanisms of how social support moderates the impact of family educational resources on BPFs in children in difficulty. Although the importance of educational resources has been widely noted, considering the generally disadvantaged position of families of children in difficulty, the present study focused on a given condition—the need for family educational resources for children in difficulty. The study explored how social support moderates the effect of educational resources on BPFs: that is, across different social supports, the effect of family educational resources on BPFs differs in magnitude. Specifically, we conducted an in-depth analysis of the relationship between positive social support and BPFs in children in difficulty with home educational resources, and the relationship between negative social support and BPFs in children in difficulty under the same conditions.

### 5.1. Negative Social Support for Children in Difficulty Whose Families Have Resources for Education

Children in difficulty are often at the margins of society, and their situation is closely linked to a high risk of developing BPFs. For people with BPFs, their disadvantaged position in social situations is often directly linked to their perceived lack of social support (Beeney et al., 2018). Previous studies have shown that BPFs are closely associated with a lack of social support, frequently experienced negative interpersonal interactions, and lower levels of social integration (Bijttebier & Vertommen, 1999; Beeney et al., 2018). These intertwined factors combine to increase the risk of BPFs among children in difficulty and undermine the positive role of home-based educational resources, such as desks, in promoting the social inclusion of children in difficulty. Globally, children in difficulty face serious challenges in accessing the necessary psychosocial support. Although social support is a fundamental right of the child, it is often not recognized and guaranteed in practice. Therefore, all stakeholders in education should pay close attention to this issue and actively promote whole-person education to ensure that every child’s educational needs are met (Mathwasa et al., 2022). To reduce the risk of developing BPFs for children in difficulty due to a lack of educational resources at home, a series of effective measures must be adopted that are based on the child’s self-awareness and active participation. Specifically, it is crucial to enhance the perception of social support among children in difficulty, which helps them realize that they are not alone but rather are cared for and supported by society. Through such measures, children in difficulty can establish positive social participation, which not only helps reduce their risk of developing BPFs but also promotes their all-around development in all aspect.

### 5.2. Positive Social Support for Children in Difficulty Whose Families Have Resources for Education

The emergence of BPFs in children in difficulty is the result of many multidimensional factors. Therefore, the family and the social environment must be considered as a whole, and the role of education in enhancing the social integration of these children needs to be promoted. Social support is an effective way for individuals to combat and regulate psychological stress, and individuals with high levels of support reveal more positive coping styles. Social support can be divided into effective formal and informal social support. Formal social support refers to support from the government, schools, medical institutions, and other formal organizations. This type of support is important in children’s mental health and BPF prevention and includes providing professional mental health services and a structured learning environment as well as developing and implementing policies and programs related to children’s mental health (Bauer et al., 2021). By contrast, informal social support refers primarily to emotional and material help in social networks such as family, friends, neighborhoods, and communities. Such support is indispensable to children’s mental health because of its proximity and accessibility. Informal social support can provide children with a warm and supportive environment in which to grow, helping them build positive relationships and increase resilience to stress and challenges (Liu et al., 2021). Social support, especially positive interpersonal interactions, is essential to mitigate the negative effects of life stress on an individual’s health (Cobb, 1976). Perceived social support contributes to maintaining positive emotions, reduces stress, and has a potentially critical role in improving the mental health of people with BPFs (Jackson et al., 2000; Thadani et al., 2022). When children face adversity, supportive social relationships can significantly mitigate its impact on their development of borderline personality traits (Elzy, 2011). If positive social support is received, it can partly compensate for the lack of family education resources, protect the emotional and psychological health of children in distress, and alleviate the intensification of BPFs brought about by the lack of family education resources for children in difficulty.

Therefore, to reduce the levels of BPFs among children in difficulty, social support must work properly, and it must be considered and designed in depth on multiple levels, including policy and culture. The policy level should provide a framework and guidance on access to educational resources for children in difficulty while ensuring that children in difficulty have access to the necessary medical care, legal assistance, and mental health services. In addition, an effective social support system should be able to integrate the resources of the family and the school into a comprehensive care program, strengthen the support network of the family and the community, improve children’s perception of social support, and provide children in difficulty with more considerate support. The material and emotional support that children in difficulty can receive from the government, teachers and peers in schools, families, communities, and others can be used as a resource supplement to help children in difficulty cope effectively with the problems caused by insufficient resources for family education and reduce negative emotions. Through this integrated approach, a more inclusive and supportive environment is created for children in difficulty, and their psychological resilience and social adaptability are promoted, thereby reducing the risk of developing BPFs and helping them to better integrate into society.

### 5.3. Limitations and Future Studies

This study identified an easily observable association between a proxy indicator, an independent study desk at home, and BPFs in children. Children in difficulty who have educational resources show a lower risk of developing BPFs. Additionally, the study found that social support had a significant moderating effect on the pathway of desk and BPF formation, providing empirical support for the study of BPFs in children in difficulty in a specific sociocultural context in China. However, this study also has limitations that could be addressed in future research. First, the cross-sectional data collection in the study was limited to children in difficulty in Hubei Province, China, and did not cover a broader national sample, limiting the generalizability of the findings. Future research could conduct longer-term, more scientific, and detailed social surveys in more countries and regions as well as qualitative in-depth interviews to further explore the relationship between the environment, home education resources, and BPFs. Second, the PSM approach reduces the reliance on a specific functional form by constructing a counterfactual framework and can partly mitigate selection bias. However, it is limited by observational selection, does not entirely address endogenous problems such as self-selection and omitted variables, and is insufficient to fundamentally address problems caused by selection bias or omitted variables. We have endeavored to consider a wide range of factors influencing the borderline personality of children in difficulty from an environmental perspective, but some important variables may not have been adequately considered. Third, our study is a large-scale self-reported survey using self-administered or guardian-completed questionnaires as the data collection method. All questionnaires used in our study were self-report measures, and the data source was children in difficulty only; hence, the data are susceptible to children’s memory biases, personal perceptions, and expectations. Future studies could consider recruiting families of children in difficulty and collecting data separately for the entire family; this would improve the accuracy of the data and help determine the impact of family and environment on BPFs in children. Fourth, borderline personality is more often revealed after adolescence, and whether the process from childhood to adolescence will result in changes in an individual’s borderline personality is uncertain due to a variety of personal, family, school, and social factors. This study examined the association between the lack of family educational resources and borderline personality in the context of social support and school climate. However, long-term follow-up experiments or social work intervention studies have not been conducted to determine long-term trends. The details of the practical process need to be further explored, which is a direction in which future research could be conducted. Our findings may have important theoretical and practical significance, and we hope that they will lead to more social research on the psychological conditions of children in difficulty, especially those with BPFs. A particular focus should be on the role of social support and family care in compensating for the lack of family educational resources and regulating the borderline personality of children in difficulty.

## 6. Conclusions

Helping people in difficulty integrate into society is one of the services and goals of social work. Rather than focusing solely on individual-level risk factors such as children’s psychological vulnerability and behavioral difficulties, the present study adopted a broader, ecological perspective by examining proximal environmental conditions, specifically the availability of material educational resources in the home, as contextual correlates of BPFs in children in difficulty. Societies consist of individuals, and communication and integration between individuals are essential for social cohesion. With the growing influence of social networks on daily life, the ability to integrate socially will be increasingly significant in the development of individuals and societies. Therefore, attaching importance to and promoting the social integration of children in difficulty and mobilizing more social forces to participate in the process are key ways to safeguard children’s welfare and improve their well-being. Although the objective presence of hidden factors has different operational definitions related to an individual’s perception of the environment (Elzy & Karver, 2018), the extent to which adversity influences an individual’s growth may vary somewhat (Jayawickreme et al., 2021). However, help for vulnerable groups, such as children in difficulty, should not be limited to improving their conditions but should also aim to use external resources to improve their maladaptive environments. Additionally, when treating individuals with BPFs, more attention should be paid to their environment (Ditrich et al., 2021; Lee & Choi, 2023). We hope to better understand the unfavorable associations between the lack of family education resources and the social integration of children in difficulty by making improvements at the social level. We look forward to conducting further research on BPFs and social integration so that more social forces can be involved in the social integration of children in difficulty and work together to create more favorable conditions for their healthy growth and all-around development.

## Figures and Tables

**Table 1:** Descriptive characteristics (mean/observations) of participants, grouped by desk, before and after propensity score matching.

Variables	Total	Unmatched		Matched	
		Without Desk	With Desk	Without Desk	With Desk
Age (9–18)	12.52 (2.32)	12.39 (2.26)	12.55 (2.33)	12.41 (2.26)	12.43 (2.28)
Hukou					
Countryside	12,300 (82.04%)	2537 (88.09%)	9763 (80.60%)	1961 (86.92%)	1875 (86.89%)
City	2693 (17.96%)	343 (11.91%)	2350 (19.40%)	295 (13.08%)	283 (13.11%)
Gender					
Female	7469 (49.82%)	1370 (47.57%)	6099 (50.35%)	1111 (49.25%)	1056 (48.93%)
Male	7524 (50.18%)	1510 (52.43%)	6014 (49.65%)	1145 (50.75%)	1102 (51.07%)
Father’s education (1–6)	2.19 (0.70)	1.92 (0.57)	2.26 (0.72)	1.99 (0.57)	1.97 (0.59)
Mother’s education (1–6)	2.20 (0.71)	1.93 (0.58)	2.26 (0.72)	2.00 (0.58)	1.97 (0.60)
Siblings	0.69 (0.80)	0.78 (0.85)	0.66 (0.79)	0.76 (0.82)	0.77 (0.88)
School life (1–4)	2.64 (0.44)	2.54 (0.48)	2.66 (0.42)	2.58 (0.47)	2.58 (0.45)
Caregiver’s parenting (1–4)	3.28 (0.35)	3.12 (0.37)	3.32 (0.33)	3.17 (0.35)	3.17 (0.34)
Parental relationship (0–2)	0.99 (0.92)	0.90 (0.87)	1.01 (0.93)	0.91 (0.88)	0.90 (0.91)
Social support (1–5)	4.57 (0.75)	4.37 (0.85)	4.62 (0.72)	4.45 (0.81)	4.44 (0.83)
DASS (1–4)	1.29 (0.49)	1.41 (0.55)	1.26 (0.47)	1.37 (0.53)	1.36 (0.54)
Observations	14,993	2880	12,113	2256	2158

**Table 2:** Balancing hypothesis test.

Variables	Unmatched	Mean		Bias (%)	*t*-Value	*p*-Value
	Matched	Treated Group	Control Group			
Age	U	−0.003	−0.074	7.2	3.43	0.001
	M	−0.004	−0.048	4.5	3.46	0.001
Hukou	U	0.194	0.119	20.7	9.44	<0.001
	M	0.190	0.191	−0.3	−0.21	0.831
Gender	U	0.497	0.524	−5.6	−2.68	0.007
	M	0.497	0.491	1.2	0.97	0.334
Father’s education	U	0.091	−0.379	51.6	23.34	0.000
	M	0.070	0.028	4.7	3.41	0.001
Mother’s education	U	0.088	−0.365	49.4	22.44	<0.001
	M	0.067	0.012	5.9	4.33	<0.001
Siblings	U	−0.036	0.113	−14.7	−7.25	<0.001
	M	−0.035	0.027	−6.1	−4.65	<0.001
School life	U	0.051	−0.215	25.8	12.89	<0.001
	M	0.048	0.131	−8.0	−6.25	<0.001
Caregiver’s parenting	U	0.117	−0.451	57.2	28.63	<0.001
	M	0.112	0.143	−3.1	−2.54	0.011
Parental relationship	U	0.013	−0.105	12.1	5.73	<0.001
	M	0.009	0.018	−1.0	−0.73	0.465
Social support	U	0.072	−0.250	30.9	15.75	<0.001
	M	0.070	0.045	2.4	2.09	0.037
DASS	U	−0.067	0.234	−29.1	−14.73	<0.001
	M	−0.065	−0.055	−1.0	−0.87	0.384

Note. U = Unmatched, M = Matched.

**Table 3:** Average treatment effect of having a desk on adolescents’ BPFs.

Treatment	Estimation	Bootstrap Std. Error	*z*	*p* > *z*	[95% Confidence Interval]	
Desk	−0.113	0.03	−3.78	<0.001	−0.172	−0.055

Note. Standard error was estimated by bootstrapping (repeating 500 times).

**Table 4:** Robustness checks.

Methods	Estimation	Std. Error	*z*	*p* > *z*	[95% Confidence Interval]	
Nearest-neighbor matching	−0.046	0.020	−2.31	0.021	−0.085	−0.007
Kernel matching	−0.082	0.028	−2.9	0.004	−0.137	−0.026
Local linear matching	−0.072	0.026	−2.73	0.006	−0.123	−0.020

**Table 5:** Moderating effects of four potential positive support sources.

Variables	DV: BPFs		
	Model 1	Model 2	Model 3
Desk	−0.077 ***	−0.085 ***	−0.079 ***
	(0.010)	(0.010)	(0.010)
IV × Social support		−0.043 ***	
		(0.008)	
IV × School life			−0.008
			(0.009)
Age	−0.007 ^†^	−0.007 ^†^	−0.007 ^†^
	(0.004)	(0.004)	(0.004)
Hukou	0.006	0.006	0.006
	(0.010)	(0.010)	(0.010)
Gender	−0.002	−0.002	−0.002
	(0.007)	(0.007)	(0.007)
Father’s education	0.003	0.003	0.003
	(0.014)	(0.014)	(0.014)
Mother’s education	0.001	0.001	0.001
	(0.014)	(0.014)	(0.014)
Siblings	0.000	0.000	0.000
	(0.004)	(0.004)	(0.004)
School life	−0.018 ***	−0.019 ***	−0.012
	(0.004)	(0.004)	(0.008)
Caregiver’s parenting	−0.096 ***	−0.096 ***	−0.096 ***
	(0.004)	(0.004)	(0.004)
Parental relationship	0.001	0.001	0.001
	(0.004)	(0.004)	(0.004)
Social support	−0.034 ***	−0.002	−0.034 ***
	(0.005)	(0.007)	(0.005)
DASS	0.128 ***	0.128 ***	0.128 ***
	(0.005)	(0.005)	(0.005)
_cons	0.586 ***	0.594 ***	0.588 ***
	(0.010)	(0.010)	(0.010)
*N*	14,993	14,993	14,993
VIF	1.37	1.78	1.73
B-P/C-W Test *p*	<0.001	<0.001	<0.001
*R* ^2^	0.188	0.189	0.188
adj. *R*^2^	0.187	0.189	0.187

*Note*. Standard errors in parentheses. ^†^ *p* < 0.1, * *p* < 0.05, ** *p* < 0.01, *** *p* < 0.001.

## Data Availability

The data are not publicly available due to privacy restrictions, as they include responses from human participants.
